# *‘We all want to succeed, but we’ve also got to be realistic about what is happening’*: an ethnographic study of relationships in trial oversight and their impact

**DOI:** 10.1186/s13063-017-2305-9

**Published:** 2017-12-22

**Authors:** Anne Daykin, Lucy E. Selman, Helen Cramer, Sharon McCann, Gillian W. Shorter, Matthew R. Sydes, Carrol Gamble, Rhiannon Macefield, J. Athene Lane, Alison Shaw

**Affiliations:** 1 MRC ConDuCT Hub for Trials Methodology Research, Population Health Sciences, University of Bristol, Bristol, UK; 2 Bristol Randomised Trials Collaboration, University of Bristol, Bristol, UK; 3 Formerly: Health Services Research Unit, University of Aberdeen, Aberdeen, UK; 4 Psychotraumatology, Mental Health and Suicidal Behaviour, Psychology Research Institute, Ulster University, Belfast, UK; 5 School of Health and Social Care, Teesside University, Middlesbrough, UK; 6 MRC Clinical Trials Unit at UCL, London, UK; 7 MRC London Hub for Trial Methodology Research, London, UK; 8 MRC North West Hub for Trials Methodology Research, Institute of Translational Medicine, University of Liverpool, Liverpool, UK

**Keywords:** Randomised controlled trials, Trial oversight, Trial steering committee, Trial management, Ethnography, Qualitative research

## Abstract

**Background:**

The oversight and conduct of a randomised controlled trial involves several stakeholders, including a Trial Steering Committee (TSC), Trial Management Group (TMG), Data Monitoring Committee (DMC), funder and sponsor. We aimed to examine how the relationships between these stakeholders affect the trial oversight process and its rigour, to inform future revision of Good Clinical Practice guidelines.

**Methods:**

Using an ethnographic study design, we observed the oversight processes of eight trials and conducted semi-structured interviews with members of the trials’ TSCs and TMGs, plus other relevant informants, including sponsors and funders of trials. Data were analysed thematically, and findings triangulated and integrated to give a multi-perspective account of current oversight practices in the UK.

**Results:**

Eight TSC and six TMG meetings from eight trials were observed and audio-recorded, and 66 semi-structured interviews conducted with 52 purposively sampled key informants. Five themes are presented: (1) Collaboration within the TMG and role of the CTU; (2) Collaboration and conflict between oversight committees; (3) Priorities; (4) Communication between trial oversight groups and (5) Power and accountability. There was evidence of collaborative relationships, based on mutual respect, between CTUs, TMGs and TSCs, but also evidence of conflict. Relationships between trial oversight committees were influenced by stakeholders’ priorities, both organisational and individual. Good communication following specific, recognised routes played a central role in ensuring that relationships were productive and trial oversight efficient. Participants described the possession of power over trials as a shifting political landscape, and there was lack of clarity regarding the roles and accountability of each committee, the sponsor and funder. Stakeholders’ perceptions of their own power over a trial, and the power of others, influenced relationships between those involved in trial oversight.

**Conclusions:**

Recent developments in trial design and conduct have been accompanied by changes in roles and relationships between trial oversight groups. Recognising and respecting the value of differing priorities among those involved in running trials is key to successful relationships between committees, funders and sponsors. Clarity regarding appropriate lines of communication, roles and accountability is needed. We present 10 evidence-based recommendations to inform updates to international trial guidance, particularly the Medical Research Council guidelines.

## Background

The oversight of clinical trials aims to safeguard study participants, and ensure that trials are run in accordance with Good Clinical Practice (GCP), and trial data are of high calibre (i.e. reliable) and collected in a reasonable timeframe. In the United Kingdom (UK), the Medical Research Council (MRC) guidelines for Good Clinical Practice (1998) define a three-committee trial oversight structure for MRC trials and this approach has been broadly adopted in the UK [1]. These committees are: a Trial Management Group (TMG) that oversees the day-to-day running of the trial, a Data Monitoring Committee (DMC) that reviews accumulating data and a Trial Steering Committee (TSC), with independent members, that provides oversight and supervision [2]. The MRC guidelines also acknowledge additional stakeholders: the funding bodies which pay for trials, and the sponsors that take responsibility for initiating, managing, and/or financing the trial and ensuring that appropriate standards are met [2, 3].

As trial methods develop, oversight guidance also requires updating [1, 4–6]. Academic Clinical Trials Units (CTUs) have come to play an increasingly important trial oversight role, working within TMGs and TSCs. Although the MRC guidelines explicitly mention ‘MRC Units’ and ‘Trials Offices’, they do not refer to CTUs. CTUs are specialist units with a specific remit to work with the leading investigators to design, conduct, analyse and publish clinical trials and other studies [7]. CTUs employ staff with expertise in trial design, trial coordination and statistics, and sometimes in qualitative research and health economics, who support trials through to completion. There has also been a change in terminology: the MRC guidelines defined ‘Principal Investigator’ (PI) as the person responsible for initiating the trial and running it on a day-to-day basis, including managing the budget, ensuring clear lines of communication, analysis, reporting and dissemination [2]. However, in practice these responsibilities are rarely discharged by one person; ‘Chief Investigator’ has become the generally preferred term for the trial lead [8], with the term ‘PI’ relating to the person(s) responsible for running the trials in the trial sites or leading the CTU activities. We use these definitions here.

There is also growing empirical evidence that trial guidance requires updating in response to advances in trial planning and conduct [1, 4–6]. In 2005, the DAMOCLES study of the role of DMCs in trial oversight found that the lines of communication recommended in the guidelines (the DMC reporting to the TSC and, when appropriate, to the funder) were not born out in practice: the DMC Chair often reported the recommendations of the DMC to the CI, TSC or sponsor [4]. In a recent survey of registered CTUs, Conroy et al. found much heterogeneity in the implementation of trial oversight processes and confusion regarding the diverse roles of stakeholders [1]. An expert panel which discussed the roles and responsibilities of trial oversight committees raised concerns regarding the role of funders in appointing independent TSC members [5]. Similarly, tensions and ambiguities in the roles expected of TSCs and attributes valued in TSC members can impact the TSCs’ quality assurance role [6].

Clear, updated guidelines on trial oversight which reflect current practices and requirements are, therefore, needed. Research has highlighted the complexity of relationships between trial oversight groups and the primary role of those relationships in enabling quality trial oversight [4, 5]. However, despite the impact of relationships on the quality of trial oversight and conduct, no previous research has explored these relationships or their impact on trial oversight. We therefore aimed to examine the relationships between all the stakeholders involved in trial oversight (TSC, TMG, CTU, DMC, sponsor and funder) and how they affect the trial oversight process, to inform future international guidelines.

## Methods

We conducted a cross-sectional ethnographic study [9] to explore relationships between trial oversight groups and their impact, using overt non-participant observation of TSC and TMG meetings and interviews with independent and non-independent TSC members, trial sponsors, funders and CIs. This approach is highly appropriate in exploring relationships and group working, as the data generated relates to what occurs in practice as well as what is reported to occur, and findings from observations could be explored further in the interviews. Findings from observation of TSC and TMG meetings were triangulated [10] and integrated with interview findings to give a rich, multi-perspective account. The methods have been reported previously and are summarised here for completeness [6]. Our research is situated within a post-positivist, minimally realist paradigm appropriate to applied health services research [11].

### Sample and setting

Sampling was guided by the selection of ‘information-rich cases’ that would provide insights to meet our study aim [12]. First, we selected eight randomised controlled trials (RCTs) which had a challenge to address, e.g. recruitment issue or protocol amendment. Relationships between trial oversight groups are more clearly revealed in trials experiencing challenges, as decisions need to be made on how to proceed. All included trials had a routine TSC meeting planned within the study period (March 2013 – January 2014). To help ensure representativeness, the RCTs covered a range of clinical topics, settings, interventions and oversight committee structures. RCTs were identified through the UKCRC Registered Clinical Trials Unit Network [13] and a major UK funder of trials.

Second, we used purposive sampling to select for interview independent and non-independent TSC members with possible differing perspectives on, and involvement in, the selected RCTs. Independent TSC members included TSC Chairs and Patient and Public Involvement (PPI) representatives; non-independent members included TMG members such as members of the CTU. Data collection continued for each trial until data saturation was reached, i.e. no new themes emerged during data analysis [14]. To gain wider perspectives, supplementary interviews were conducted with representatives of a purposive selection of trial funders, sponsors and CIs with experience of other challenging trials.

### Data collection

All observational and interview data were collected by the same female academic (AD), an experienced qualitative researcher with a PhD in health services research who was not previously known to the participants. The research was presented as an ethnographic study of trial oversight.

#### Observational data

For each trial, the researcher attended, observed and audio-recorded one TSC meeting and relevant TMG meetings during the study period. Field notes were taken during and after the meetings, guided by a standardised observation schedule (Table 1) developed on the basis of established guidance [9], prior studies of trial oversight [4] and our research questions.

**Table 1:** Observation schedule (summary)

Topic	Field notes
Data collection	Date, location, observer
Pre-meeting	Setting (including diagram of room layout) Arrivals (e.g. who arrives when, what order?) Pre-meeting talk (e.g. verbal and non-verbal communication)
Main meeting	Start time, who starts/how? Organisation of meeting (e.g. role of Chair, agenda, hand-outs, atmosphere) Chair (e.g. manner of facilitating discussion, leadership style, management of tension/conflict, influence on decision-making) Content of discussion (e.g. issues raised, knowledge/information drawn upon, recommendations made) Group interactions and decision-making (e.g. concerns raised, individuals’ contributions and roles in discussion, verbal and non-verbal communication, decision-making) Mode of participation (e.g. via teleconference) Action points/tasks (e.g. what is to be done, by whom, by when?) Time interval to next meeting End time, length of meeting, who ends/how?
Post-meeting	Interactions and behaviour (e.g. who leaves first, alone/together?) General impressions

#### Interview data

TSC members were invited for interview by the relevant trial CI or by the study researcher. Interviews were conducted before or after the TSC meeting depending on availability and preference. Supplementary interviews were conducted with funder, sponsor and CI representatives, identified via snowball sampling [15]. Interviews were face-to-face or by telephone, depending on participant preference, and were recorded, transcribed verbatim and checked by the researcher prior to analysis. One interview was with two people (two sponsor representatives, #5 and #6), the rest were conducted individually.

Semi-structured interview topics guides (summarised in Table 2) were formulated based on the academic literature regarding trial oversight and the research team’s expertise.

**Table 2:** Interview topic guides (summary)

Participant groups	Topics discussed in interviews
Chief Investigator, Trial manager	The trial: history of the trial, details of the trial, current stage, successes, current and anticipated challenges The Trial Steering Committee/Trial Management Group (TSC/TMG): expectations of TMG/TSC, composition of TMG/TSC, selection of members and chairs, nature of the group’s decision-making and members’ involvement, examples of actioned group recommendations, impact of TMG/TSC, communication between TSC and TMG, relationship and communication between trial oversight committees and with funder
TSC members	The trial: history of participation in the TSC, views of TSC, relationships with other members, value of TSC meetings, TSC’s role in decision-making, relationship and communication between TSC, TMG and other trial oversight committees TSC meetings: meeting organisation, Chair and leadership, communication during meeting, decision-making, agreeing and assigning actions, communication of actions to other groups/trial personnel
TMG members	The trial: history of participation in the TMG, views of TMG, relationships with other members, views of TMG and TSC, value of TMG/TSC, TMG/TSC role in decision-making, relationship and communication between TSC, TMG and other trial oversight committees TMG meetings: meeting organisation, Chair and leadership, communication during meeting, decision-making, agreeing and assigning actions, communication of actions to other groups/trial personnel, role of Patient and Public Involvement (PPI)
Trial funders	Funders’ expectations and views of TMGs/TSCs, process of selecting TSC, examples of trial oversight working well, examples where trial oversight has not worked well, different models of TSCs, role of TSC Chair, role of PPI, role of the trial funder, regulatory bodies, recommendations
Sponsors	Sponsors’ expectations and views of TMGs/TSCs, role of sponsor in trial, responsibilities of sponsor, relationship between trial oversight committees, sponsor and funder, challenges faced by trials, role and value of TMGs/TSCs

*TMG* Trial Management Group, *TSC* Trial Steering Committee, *PPI* Patient and Public Involvement

### Data analysis

Observational data (meeting recordings and field notes) and interview data were analysed thematically [16]. Analysis by the primary researcher (AD) occurred alongside data collection, to enable emerging findings to inform subsequent data collection. An initial coding framework was developed using techniques of constant comparison [17] and a combination of deductive line-by-line coding, based on the research aims, and inductive analysis. The primary researcher (AD) met with members of the research team (AS, SM, GS and HC) monthly to review data analysis and emerging findings. During the meetings, the coding frame was refined, and data scrutinised for disconfirming and confirming perspectives. AD coded all the data. All the interview and observational data from four of the RCTs were also independently coded by another researcher (AS, SM, GS or HC). Identified minor differences were discussed by the team, resolved and integrated into the analysis. Finally, a narrative summary of the findings, which integrated data from the interviews and observations, was constructed (LS, AD). Triangulation attended to divergence and convergence in the datasets and the different perspectives represented [9]. Data analysis was managed in NVivo v.10 [18].

Data have been anonymised to protect confidentiality, with ID codes and trial number used to identify quotes from interviews. Observational data from meeting recordings and field notes are assigned to a trial number.

### Ethical approval

The University of Bristol Faculty of Health Sciences Research Ethics Committee approved the study. National Health Service (NHS) research governance approval was gained where data collection took place on NHS premises or included NHS staff. All interview participants gave written informed consent.

## Results

### Data collected

We observed and audio-recorded eight TSC and, six TMG meetings (range 40–120 min in length). We conducted 66 interviews with 52 individuals. Thirty were TMG members for the included trials, 14 were independent TSC members, and the remainder were other relevant informants working in this field (*n* = 8) (Table 3). All those approached agreed to be interviewed. Interviews ranged from 19 to 148 min (mean 58 min). The median number of interviews per trial was 10, range 6–11.

**Table 3:** Characteristics of interview participants

Participant ID	Role	Relationship to trial	Gender	Trial number	Trial subject area
01	TSC Chair (clinician)	Independent	M	1, 2	Oncology
02	Senior trial project lead	TMG	M	1, 5	Oncology
03	TSC coordinator P1	TMG	M	1, 2	Oncology
04	TSC coordinator P2	TSC administrator	F	1, 2	Oncology
05	Sponsor representative	TMG	F	1, 2	Oncology
06	Sponsor representative	Observer at TSC meeting	M	1, 2	Oncology
07	Trial manager	TMG	F	1	Oncology
08	CI	TMG	F	2	Oncology
09	Trial manager	TMG	F	2	Oncology
10	Trial manager	TMG	M	3	Arthritis
11	Senior statistician	TMG	M	3	Arthritis
12	Senior trial manager	TMG	F	3	Arthritis
13	Statistician	TMG	M	3	Arthritis
14A	Co-CI^a^	TMG	M	3	Arthritis
15	TSC Chair (clinician)	Independent	M	3	Arthritis
16	Trial manager	TMG	F	4	Frailty
17	TSC Chair (methodologist)	Independent	M	4	Frailty
18	CI	TMG	M	4	Frailty
19	TMG Chair	TMG	F	4	Frailty
20	TMG member	TMG	F	4	Frailty
21	Trial manager	TMG	F	5	Oncology
22	Statistician	TMG	F	5	Oncology
23	CI	TMG	M	5	Oncology
24	TSC member	Independent	M	5	Oncology
25	TSC member	Independent	M	5	Oncology
26	Trial manager	TMG	F	6	Urology
27	Trial manager	TMG	F	6	Urology
28	Statistician	TMG	M	6	Urology
29	CI	TMG	M	6	Urology
30	TSC Chair (clinician)	Independent	M	6	Urology
31	TSC member	Independent	M	6	Urology
32	TMG member	TMG	M	6	Urology
33	Trial manager	TMG	F	7	Psychology
34	CI	TMG	F	7	Psychology
35	PPI Representative	TMG	M	7	Psychology
36	TSC Chair (clinician)	Independent	F	7	Psychology
37	TSC member (statistician)	Independent	M	7	Psychology
38	TSC member	Independent	M	7	Psychology
39	CTU Director	TMG	F	7	Psychology
40	Senior trial manager	TMG	F	7	Psychology
41	Trial manager	TMG	F	8	Oncology
42	TSC Chair (clinician)	Independent	M	8	Oncology
43A	PPI representative	Independent	F	8	Oncology
43B	PPI representative	Independent	M	8	Oncology
44	Senior statistician	TMG	M	8	Oncology
45	Sponsor representative	Independent	M	8	Oncology
46	CI of another trial/member of TSCs	n/a	M	n/a	n/a
47	Funder representative	n/a	M	n/a	n/a
48	Sponsor representative	n/a	M	n/a	n/a
49	Funder representative	n/a	F	n/a	n/a
50	Senior statistician	n/a	F	n/a	n/a
51	Funder representative	n/a	F	n/a	n/a

^a^Note: the Co-CI from trial 3 (14B, male) was not interviewed, but was recorded during a meeting (Table 4)

*CI* Chief Investigator, *TMG* Trial Management Group, *TSC* Trial Steering Committee

### Findings

Five main themes regarding relationships between the stakeholders involved in trial oversight were identified: (1) Collaboration within the TMG and role of the CTU; (2) Collaboration and conflict between oversight committees; (3) Priorities; (4) Communication between trial oversight groups and (5) Power and accountability (subthemes: 5a. Perceived power and accountability; 5b. Impact of perceived power on relationships).

#### Collaboration within the TMG and role of the CTU

Evidence of collaborative relationships between those involved in delivering trials demonstrated the benefits of trial teams bringing together experts with complementary skills who collaborate with mutual respect. PPI members brought an awareness of the patient participant perspective (and their in-trial conduct will be explored in detail in a separate publication):

*‘My actual role, as I see it, is to represent the patients as much as I possibly can, to make sure their interests are dealt with correctly, that they are not just used as pawns in the research’*. [#35, PPI representative, RCT 7]

CTU members, who rarely had a clinical background, relied on the CI for subject expertise, while CIs relied on the expertise of the TMG, TSC and CTU to advise on research processes, and trial science, conduct and regulation:

*‘Things work well in trials in general when you’ve got the clinician side who are on top of all of the kind of treatments and that kind of thing, and then you’ve got us lot here who make it all work. The clinicians don’t always understand how much time it takes to put in amendments and how much trouble it can cause at sites… But without them, obviously there’d be no trial’*. [#41, Trial manager (TM), RCT 8]

*‘We look to [CIs] to make sure that we are understanding a lot of the background and the issues in clinics. We have to make sure that [they] understand the issues relating to the design of the operational aspects of the study.’* [#02, Senior trial project lead, RCTs 1 and 5]

The TMG meeting for trial 5 was an example of a collaborative and productive exchange between CI and CTU lead. The CI introduced what he considered to be minor changes to trial data collection:

*‘So what we wanted the group to approve was just formal collaboration with [another researcher], which seems to be pretty straightforward. Yes, they just want to take some extra [tissue] samples from patients in the trial, they don’t want to do anything to them, apart from that. So I just formally wanted to request the TMG approve this’*. [#23, CI, RCT 5]

The CTU senior lead guided the CI through the reasons why the proposed changes would require a protocol amendment and both parties agreed a way forward:

*‘Okay, so we need to think about what the implications are. What data they are looking for, whether this is data that we would be able to get out of the centres. Were the centres going to struggle to collect that information? Whether they want this data released, what the impact is for us? I guess this another protocol amendment?’* [#2, Senior project trial lead, RCTs 1 and 5]

*‘Yes.’* [#23, CI, RCT 5]

*‘So it’s not a small amount of work required, by our end I suspect.’* [#2, Senior project trial lead, RCTs 1 and 5]

Fundamental to good collaboration in this case was openness to benefitting from the knowledge and skills of others. CTU staff reported their advisory role was easier if CIs were open to learning and collaboration, but this depended on the CI’s personality:

*‘They don’t need to understand it [all of the operational details of running a trial], so long as they are the kind of person who can admit, “Okay, I don’t understand this and so you’re going to advise me”… But I have worked with some CIs who are a little bit more difficult and who maybe don’t understand how trials work and try and argue sometimes’*. [#41, TM, RCT 8]

Trial managers (TMs), usually employed by a CTU, were key in supporting the CI by running day-to-day trial management. TM tasks included keeping an eye on deadlines and recruitment targets, arranging TSC and TMG meetings, and coordinating or implementing decisions made:

*‘I probably have the most input in the day-to-day management of the trial, so I update on trial progress. I would also usually be responsible for implementing the decisions that are made’*. [#33, TM, RCT 7]

*‘It is very much a project management role when it gets to this stage: keeping an eye on deadlines and making sure that we achieve our targets.’* [#27, TM, RCT 6]

TMG meetings were felt to work best when they were frequent enough to deal quickly with emerging challenges, with the necessary frequency dependent on the trial:

*‘You’ve got weekly meetings with a sort of smaller group of people, that’s the agility you need to make decisions, even once a month to be honest is probably not quite agile enough for a TMG’.* [#31, Independent TSC member, RCT 6]

Where relationships between CI and TM worked well and CIs were confident in the trial team, TMs facilitated the research process and supported the CI by providing day-to-day management of the trials. The CIs appreciated this:

*‘When you are working with a good team, it is not that stressful, there is a lot of satisfaction from it’*. [#29, CI, RCT 6]

#### Collaboration and conflict between oversight committees

There was evidence of how the TSC delivered good trial oversight and guarded the rigour of the trial by raising for discussion key features of trial progress, such as recruitment across sites and to different trial arms, whether to release interim data, and statistical analysis plans. Reaching and delivering recommendations to improve trial conduct involved collaboration and discussion between trial oversight committees. For example, trial 2 had been threatened with closure due to poor recruitment, and the TSC had taken the *‘highly unusual’* (#08, CI, RCT 2) step of authorising an interim release of data to reassure clinicians recruiting to the trial that there had been no safety issues to date. This involved the TSC, DMC and TMG reaching consensus, and resulted in the trial being kept open, as the CI explained:

*‘It has been slow to recruit, but it is continuing to recruit. The TSC and the TMG at the [funder] have been very innovative, sensible and imaginative in trying to stretch our funding. [Interviewee laughs]… What was clear from the interim release was that we did not appear to be doing harm. That boosted recruitment and it encouraged clinicians to know that it was worth having the conversation: “I don’t seem to be doing harm with this trial, and it remains a very important question to answer”. That allowed us to maintain our funding and to stretch it’.* [#08, CI RCT 2]

In trial 6, however, the CI saw TSC meetings predominantly as a *‘rubber stamping process’* (#29, CI, RCT 6) and questioned the value of the TSC to the trial. This was in contrast to the TM and senior statistician, who felt that the TSC had been valuable in improving data return rates. The TSC Chair himself reflected on how the TSC wasn’t currently influencing the trial *‘because it’s going quite well’* (#30, TSC Chair, RCT 6), suggesting collaboration is most needed when problems arise:

*‘There wasn’t very much on the agenda really, it’s rather formulaic in that we always, you know, the recruitments and the questionnaires and how many have been filled in and then well what the DMC have said and how the thing is just going generally, because it’s going quite well, there isn’t a lot to say really. So it’s… we have to hold the meeting because you know that’s in the protocol really for a couple of times a year. So I don’t think we did anything or said anything earth shattering… I never know what people think about these things but they all come along, probably because they have to’*. [#30, TSC Chair, RCT 6]

Trial 7 exemplified both conflict and collaboration in trial oversight. At the time of the study, trial recruitment had been stopped due to concerns about patient safety. This decision was taken after the DMC noted a safety signal due to increased hospitalisations in one of the trial arms, and recommended to the TSC the trial should stop recruiting, but continue the allocated intervention with patients already randomised. The TSC considered the advice of the DMC and asked that both the recruitment *and* delivery of the intervention be stopped, with only follow-up data collection continuing to ascertain reasons participants had sought help. During this process, the TSC asked to be unblinded to the data and requested that the DMC stand down. The TSC’s decision to cease recruitment, informed by the DMC’s recommendation, was initially distressing for the CI and TMG, and there was conflict:

*‘Our position then was, as a TSC… we have taken decisions here, and we are absolutely obligated to explain those decisions. The meeting started in a quite hostile fashion, because the PIs and the CIs were just basically saying, “What the hell has happened here? What have you done?” We had to get it across to them, and it was a very good meeting. Because by the end of it, everybody was on the same page. The previously hostile PIs were actually quite grateful that the situation had been explained to them. They now had a mechanism to engage their patients’.* [#38, Independent TSC member, RCT 7]

The TMG subsequently collaborated well with the TSC to ensure that the closure of the trial was carried out optimally for the participants:

*‘What there was complete agreement about, was concern for participants and the welfare of participants in the trial… everybody had agreed on that, and it was just disagreement about how to deal with that’.* [#39, CTU Director, RCT 7]

#### Priorities

The degree to which relationships between those involved in trial oversight were collaborative and constructive was influenced by stakeholders’ priorities. These priorities, whether individual or organisational, were associated with individual’s perceptions of what was required to maintain or advance their professional reputations and careers, as well as their belief in the value of the trial. For example, CIs were often reported in interviews to be personally invested in the success of their trials in a way that other members of the TMG were not:

*‘I think he treats [the trial] as his baby, because he is the Chief Investigator… You kind of think he’s really personal about it and I think, you know, if the trial did get closed he’d be like pretty upset about it… he’s really highly motivated and driven… anything possible to keep it going’*. [#13, Statistician, RCT 3]

In contrast, members of the CTU perceived themselves to be more objective, detached and realistic about the progress of the trial:

*‘Obviously [the CI] is very close to trial and we all want to succeed, but we’ve also got to be realistic about what is happening and… I mean, as I say, I try and look at things from an external point of view as well, try and take a step back’*. [#11, Senior statistician, RCT 3]

A director of a funding body also contrasted CIs’ perceived closeness to the trial with the CTU’s objectivity, drawing attention to the CTU’s organisational priority of focussing on trials which meet recruitment and follow-up targets:

*‘The CI very, very rarely wants to have his trial closed, because it’s the sign of failure. The trials unit… can see a dead horse that they have sitting on their portfolio’*. [#51, Funder representative]

For this representative, the perceived objective nature of the CTU was part of the ‘professionalism’ central to running a trial they would define as ‘rigorous’:

*‘It should be impossible for an investigator to run a trial that isn’t done through… an accredited trials unit… There should be no investigators as they used to be when I started doing trials on the back of an envelope basically in a little office somewhere, dolling out the drugs probably – it’s not on anymore… What we are talking about now is the whole professionalism of running trials… nobody should be doing this out-with a professional trials unit, and there are many of them now… And if anybody has a halfway decent idea for a trial they need to be developing it with a trials unit’*. [#51, Funder representative]

There was consensus among respondents that the organisational priority of independent TSC members was to protect participant wellbeing while ensuring scientific quality (see [6] for further details on the role of the TSC):

*‘I think in a philosophical way, [the role of the TSC] is to protect the patient, and then the integrity of the trial… it puts the interest of the patient first and foremost. As an independent, you are making sure that that is observed’*. [#37, Independent TSC member, RCT 7]

This role was contrasted with the DMC’s prioritisation of participant safety, which one person attributed only to this committee:

*‘The safety of the patient is the exclusive reserve of the DMC’*. [#37, Independent TSC member, RCT 7]

The DMC’s primary role in trial oversight was reported to be reviewing accumulating, unblinded trial data, and, when needed, raising concerns for further consideration by the TSC, which would take the decision to act:

*‘The DMC can see the data and say there’s a problem, but they won’t know why there’s a problem… their job is to look at the data, not completely in isolation, but looking for signals, identifying the signals, passing that on to a group [the TSC] that then can look at things in more depth’*. [#40, Senior TM, RCT 7]

*‘The TSC does not have to take the DMC’s advice, because it has to see a greater picture. It almost certainly will [take DMC advice]. But every so often, the person that the subcommittee[sic] reports to should be able to turn around and say, “Well, no, based on other things that we have to take account of, your recommendation does not overwhelm”.’* [#38, Independent TSC member, RCT 7]

As independent bodies, the TSC’s and DMC’s organisational priorities were perceived as a way of keeping in check the individual priorities and/or biases other stakeholders, such as the CI or funder, might bring to trial oversight, and hence protecting participants’ wellbeing.

Differences in stakeholders’ priorities caused tension and conflict within and between trial oversight committees. For example, Table 4 presents an extract from a TMG meeting in which a Co-CI, jointly responsible for the trial, argued to change the statistical parameters of the analysis, because he feared the closure of the trial otherwise. He appealed to the trial team’s ethical duty to participants to continue with follow-up, while the TMG members urged the Co-CIs to adhere to GCP (field notes, TMG meeting trial 3). A compromise was reached prior to the TSC meeting, with the Co-CIs agreeing to reduce power from 90% to 80%. This was endorsed at the next TSC meeting.

**Table 4:** Differences in priorities causing conflict – example from RCT 3

#14B, Co-Chief Investigator (Co-CI), RCT 3: ‘So it might be also helpful [trial statistician], to look at the scenarios of seeing if there was a greater difference. I’m not suggesting that’s our primary strategy, I think the best strategy is to go for power. But I think it would be nice to have the scenarios of looking for a 70% difference and a 90% difference. So (trial statistician), would you be able to just do that?’	
#13, Statistician, RCT 3: ‘Yeah, we can do that, but when I was speaking to [senior trial statistician], yesterday he said it’s not really good practice to meddle with the difference coz you know…’	
#14B, Co-CI, RCT 3: ‘Well it’s very easy to say that but the other option is the trial stops, do you see what I mean? So good practice is to make sure that we fulfil the commitments of the families who have consented to this study. So we appreciate what good practice is, but I think it’s also looking for a number of scenarios in which we can present to everyone including the Data Monitoring Committee, the (Trial) Steering Committee and the funders to find a pragmatic solution to a problem of recruitment, okay. So it’s just to have those figures available really for discussion’.	
#12, Senior trial manager (TM), RCT 3: ‘[CI], I think it’s just being aware that obviously if you’re changing the difference that you’re looking for, then you are leaving yourself open to criticism, so you need to be ready to…’	
#14B, Co-CI, RCT 3: ‘We are –’	
#14A, Co-CI, RCT 3: ‘We are aware. [Senior trial manager], we’re open to ever greater criticism, so this is not been talked about lightly or picked from thin air. There’s a real and present danger that if we go up in front of (funder) in (month) not having met our target of [x] patients in the last 6 months, there is a very real present clear danger that they will stop the trial. That is not in the best interest of the patients who have participated, that’s not in the best interest of any of us’.	

*Co-CI* Co-Chief Investigator, *RCT* randomised controlled trial, *DMC* Data Monitoring Committee, *TSC* Trial Steering Committee, *TM* Trial manager

#### Communication between trial oversight groups

Good communication played a central role in ensuring relationships between trial oversight stakeholders were productive and the process efficient. The TM was first point of contact for the trial and often acted as the primary channel of communication between the CI and trial oversight groups. Controlling aspects of intergroup communication allowed the TM to:

*‘…make the message clear …to make sure we’re singing from the same hymn sheet…’*. [#02, Senior trial project lead, RCT 1 and 5]

*‘Communication is definitely key. It helps that all the communication goes through me. I think the TSC and (DMC) …look for emails from my email account, and they know to respond to that.’* [#10, TM, RCT 3]

The CI and TM of trial 7 attributed their previous success in dealing with a challenging issue (the early stopping of recruitment) to the clarity of communication initiated by the CTU and shared with all trial oversight parties:

*‘The most important thing is to communicate everything and expediently, and keep communications going between all groups… Transparency is important… and the relationships between the TMG, the CTU and the TSC were really vital, that’s why it worked so well’*. [#40, Senior TM, RCT 7]

Mutual respect between all members of the TMG and TSC was noted during the observation of trial 7’s TSC meeting. However, those involved in trial 7 also described a period of conflict with the trial DMC during the same challenging episode. The DMC, instead of communicating through the TSC in the expected manner, was reported to have directly contacted the TMG to gain more information:

*‘The communication issue there came up, which is, to be honest I don’t see it as my job to communicate with DMC. The route, we clarified – the route was a bit unclear way back. But during the recruitment stoppage, we were careful to clarify that the route of communication was between us and the TSC to the DMC… They advise the TSC, not us… The trouble originally was DMC badgering us’*. [#34, CI, RCT 7]

The CTU Director in this instance took on the role of arbitrator, clarifying acceptable lines of inter-group communication in trial oversight:

*‘The role of the DMC is to be independent, and they jeopardise their independence if they’re too close to the trial team – it’s not that they shouldn’t make a request for information from a TM, but it should go through some other route. The DMC reports directly to the TSC. That’s the charter that everybody uses… The first thing I did was to stop communication between the TM and the CI, and the DMC’*. [#39, CTU Director, RCT 7]

The CTU Director could take on this role as it was written into the trial’s charter. Her intervention was timely, as it prevented the process of early stoppage of recruiting going ‘pear-shaped… the CTU’s reputation was at stake’ (#39, CTU Director, RCT 7).

Clarity regarding appropriate lines of communication, a central point of contact responsible for coordinating communication, and consideration in advance of the CTU’s role as arbiter were seen as facilitators of communication between trial oversight stakeholders and trial oversight:

*‘There needs to be really, really accurate and complete communication between research team led by chief investigator and the TSC, between TSC and DMC, and these are both bi-directional not uni-directional. Then, as the need arises, between TSC and other stakeholders, which, importantly would include the funder’*. [#37, Independent TSC member, RCT 7]

#### Power and accountability

##### Perceived power and accountability

Interview participants described the possession of power over trials as a shifting political landscape. The more experienced of those interviewed talked of a time when TSCs were mostly accountable to sponsors and funders had a *‘more hands off’* approach (#39, CTU Director, RCT 7). They described how a large funder of trials had now changed its relationship with TSCs, requiring them to report directly to the funder and seeking to appoint independent TSC members itself. A representative from the funder reported that their appointment of TSC members had arisen as a protective mechanism against poor trial oversight leading to flawed trial methodology:

*‘They [the TSC] didn’t come to this with any independent scrutiny. So when a lot of cross over and, in effect, contamination between the arms was going on, the TSC were quite happy to accept that… We actually discharged the Chair of the [Trial] Steering Committee and appointed our own … experienced TSC Chair who we briefed to make it clear that what we wanted from them’.* [#47, Funder representative]

Others also recognised that public sector funders were responsible to the government for how research funds were spent, whereas the TSC’s responsibility was narrower:

*‘If you’re the funder you’ve got power and responsibility, you have still got to make sure this stuff delivers otherwise the governments are going to get upset, so you know you have got responsibility there. The TSC, yeah it has responsibilities but they are very much within … perhaps a narrower focus of responsibility, …they have a responsibility to do what they are supposed to do according to their charter. It isn’t really the TSCs responsibility if the whole thing goes wrong’*. [#31, Independent TSC member, RCT 6]

However, the funder appointing its own independent TSC members was perceived by some as the funder trying to control the trial, by shifting accountability and loyalty of the TSC from the trial to the funder. Interviewees expressed concern over this change, for example:

*‘The [funder], they make it very, very clear: it says the primary TSC reporting line is via the Chair to the [funder] programme director. Forget about the sponsor, so this is the funder who is taking control of the TSC and whereas if you look at the research governance stuff I’m sure that it was actually the sponsor who the TSC should be reporting to, not the funder…*

*They’re definitely trying to have more control of the TSC so their loyalty is more to the funder rather than to the sponsor or to the CI… I think the TSC has a very important role… it’s worrying that [funders] are trying to dilute their responsibilities or yeah just distorting the relationship by… making them much more a tool of the funder rather than a source of independent scientific advice …the TSC to my mind has become almost a puppet’*. [#50, Senior statistician]

Other participants thought the funder’s power and the TSC’s lack of power was almost inevitable given the funder’s ability to withdraw financial support:

*‘At the end of the day, the guy with the money is the guy with the power, and if the funder says do it, you’re going to do it … because they’ve got the ball. They’ll take it away if they don’t like it’.* [#31, Independent TSC member, RCT 6]

*‘The TSC is, after all, it’s an advisory group isn’t it? It's not an executive group, they don't decide on what you can and can't do and they don't really have any control over the funding. So they're there to advise the (funder), who then make the decision.’* [#29, CI, RCT 6]

This statement contradicts the charter for many TSCs, where the TSC is executive for many aspects of the trial. The question of who has overall responsibility for deciding whether a trial continues was contested by some. Some participants pointed out that a funder withdrawing financial support did not necessarily mean that the trial had to stop, even if this was likely:

*‘The question was should they have the power to stop it? I think they’ve got the power – no, the only power they’ve got is to withdraw the funding I think, because the actual protocol, as far as I’m aware, is the property of the trialists. I would have thought the trialists are able to continue the trial even if the [funder] withdraw the funding … from a pragmatic point of view, then most trials would probably stop’*. [#32, TMG member, RCT 6]

*‘No. Because they’re thinking in terms of money as opposed to whether it’s a good idea being done properly. What matters is the question whether it’s being done properly and whether you can get an answer, and the funding is just a different matter altogether.’* [#01, TSC Chair, RCTs 1 and 2]

Sponsor representatives, a funding director and two TSC Chairs all asserted that they had overall responsibility for deciding whether a trial progresses:

*‘I suppose officially it would be me really… I can’t imagine the circumstance where I would be telling [names] and the statistician and Director of [CTU] you know, this is rubbish it’s got to stop… but of course it would be my job to do that, it’s in the sort of charter’*. [#30, TSC Chair, RCT 6]

*‘The buck stops with us. They [the TSC] do slightly more than advise I guess. We would certainly look to them, I mean this is slightly confused, not confused, but there is a tripartite role really for oversight of trials because there’s us as funder, there’s the TSC employing independent advice but accountable to us as funder and the other obviously is the sponsor… the local university or hospital and they obviously have a role as well and to some extent the TSC needs to be accountable a little bit to the sponsor as well.’* [#47, Funder representative]

*‘The TSC guides the sponsor, and ultimately the decision to move forward or not with the study sits with the sponsor.’* [#45, Sponsor representative, RCT 8]

*‘[Funders] like to pretend they are but they’re not, are they. Quite clearly not, so the buck doesn’t stop with them, their role, the buck stops with the sponsor.’* [#48, Sponsor representative]

These accounts of who has the power and responsibility to stop a trial demonstrate variation in practices and processes and a significant lack of clarity about roles in trial oversight. Participants’ beliefs regarding which oversight group had the ultimate power over a trial were influenced by their working environment and allegiances, e.g. employees of one of the funders believed that sponsors had overall power, while other funder directors and their employees believed that power lay with the funders.

All these parties may be correct: the funder can stop a trial by withdrawing money and the sponsor by retracting their indemnity. The TSC Chair could no longer offer her or his support, but this role would be replaced. Each group’s reasons for stopping the trial will vary, e.g. lack of value for money, data protection breaches or patient safety. Instead of perceiving this situation to inevitably end in conflict, a CTU Director viewed it as providing ‘checks and balances’:

*‘Well, you could see it as checks and balances, and they would stop it for different reasons … I see it as just checks and balances, but then, if you’ve got a situation of conflict, what you need is a process for resolving it … It was a huge relief to me when I went and looked at the charter, because I hadn’t been involved in setting it up, so I didn’t read the charter until this happened. I was so relieved to find that there was something in there that offered a way forward … It gave me a mandate to interfere. [Laughter]’*. [#39, CTU Director, RCT 7]

In the hierarchy of power in trial oversight, the CI could be perceived to be at the bottom: despite having (co-)initiated the trial process and being clinically accountable for the trial’s success or failure, CIs were reported by one TSC member to have ‘plenty of responsibility without power’ (#31, Independent TSC member, RCT 6).

##### Impact of perceived power on relationships

Trial stakeholders’ perceptions of their own power over a trial, and the power of other stakeholders, influenced relationships between the oversight bodies. Funders were seen as *‘the overall boss’* (#38, Independent TSC member, RCT 7), with others answerable to them. Participants discussed, with trepidation, how funders contacted CIs or TMs of trials facing challenges and called the CI to monitoring meetings to account for trial progress. Several participants had experienced these meetings, while others had heard about them. The TMG of trial 3 was due its second such meeting and was still having difficulties with recruitment. During the observed TMG meeting, which the two CIs attended via teleconference, the stress caused by this was evident from their tone of voice, rapid speech, and interruptions when CTU members were talking (field notes, TMG RCT 3; see Table 4). Another participant had experienced a similar monitoring meeting; her account highlights its stressful and difficult nature:

*‘[The funders] didn’t actually call me a liar, but they said, “We don’t believe you”… which at the time made me very angry … well, it didn’t seem to me that that was a reasonable way of behaving … it wasn’t a reasoned response to the scenario that we were putting forward to them… They’d just made their minds up, they’d looked at the recruitment figures and said “Right, we’re closing this trial”… Although they initially gave the impression that it was going to be a constructive helpful discussion as to how the trial could be salvaged, they had meanwhile made the decision that they were going to close the trial before we’d actually had the opportunity to put forward our proposals for how things could be saved’*. [#50, Senior statistician]

In contrast, the funder representative called these types of monitoring meetings *‘hub visits’* and an opportunity for *‘constructive dialogue’* after the trial teams had given a presentation on the progress of their study:

*‘I mean, we don’t just see the ones that are poorly performing, although we often do call them in and talk through if there are problems and what’s going on. But it’s then an opportunity for us to sometimes provide good advice to them about how they might turn things round… and actually most people come away from those feeling they’ve had a good meeting. You know, clearly not everyone, but it’s not the sort of thing where people come up and get a bit of a kicking as it were… I mean, you do have the odd difficult one where… you basically do have to give people bad news that they’re clearly going nowhere and it’s time to close the trial down’.* [#47, Funder representative]

However, there was anxiety and fear attached to these meetings by researchers; shutting down a trial might mean the loss of opportunity to answer the research question, loss of employment due to shortened contracts, loss of reputation for the CI and the CTU, and a waste of trial oversight committee members’ time.

The CIs’ response to the perceived danger of the funder stopping an insufficiently performing trial was to try to present themselves and their trial in a positive light, primarily to the TSC, which reported directly to the funder. Some trial team members described how CIs put a favourable *‘spin’* or *‘shiny surface’* (#20, TMG member, RCT 4) on their progress reports when the trial oversight groups met, suggesting that this was either human nature in face of a threat, or the nature of academia:

*‘Because ultimately the TSC is accountable to the funder, and there is the big worry that they are the people that could stop your trial … that they would withdraw your funding. So I suppose people want to keep them sweet… Make them think you are doing a good job. Also because it is human nature, everybody does it’*. [#33, TM, RCT 7]

*‘We want to look good; we want to look confident. I think people in academia, people doing research, are people who probably tend to be on the perfectionist end of things. They want things to be nice; they want things to go well. Yes, I think people want to feel like they’re competent and in charge and on top of their game, and can do it.’* [#20, TMG member, RCT 4]

*Interviewer: ‘Mm-hmm, and so we – we don’t lie, do we?’ ‘No, I don’t think we lie. I don’t think I have ever sat here and lied, and I don’t think any of us have ever sat here and lied, but I think we put spin on things… there’s what actually has happened, and then there will be the spin we put on that to make it look like something different happened. I think we do that all the time.’* [#20, TMG member, RCT 4]

Independent members of TSCs also reported being conscious they used *‘spin’* when conducting their own trials. One such respondent suggested the trial team’s role in a TSC meeting was to give confidence to the independent members that the trial is under control. He proposed that communication which is not totally open, but ‘managed’, is at times not just justifiable but preferred:

*‘I’ve got a couple of CIs who like to go off in tangential discussions about the study and how it might be better, and you know opportunities missed. I say to them, “Could you just not do that?” It’s not the purpose, we are here to basically reassure the independent [TSC members]. Now, on the one hand I can see that, intellectually that might be not quite right, that we are there to have an open discussion… So I think the best kind of TSC is a good, compromise between those two. That there is a genuine sense of open debate and inquiry, but it’s based on rock solid data, which has been produced to a professional standard’.* [#37, Independent TSC member, RCT 7]

The use of spin was evident observationally in guarded or incomplete reporting of information by trial teams to their TSCs, when compared with previous discussions witnessed in TMG meetings (field notes, TMG and TSC meetings for RCTs 3 and 4).

However, some respondents felt experienced TSC independent members would or should see through the spin and *‘scratch the shiny surface’* (#20, TMG member, RCT 4) to gain a realistic view of trial progress:

*‘I guess the CI could try to do that, but I think if the TSC has enough independent members and is doing its job correctly, they would be willing to question and wouldn’t be led inappropriately’.* [#49, Funder representative]

*‘I would like to think that the old goats that serve on these things are long enough in the tooth to see through that rubbish.’* [#28, Statistician, RCT 6]

A CTU Director and a sponsor representative both said the trial teams, but especially the CIs, were foolish to present a shiny surface to their TSC by giving overly optimistic verbal reports, as it prevented the TSC from ensuring that the trial was well-run. Instead, they believed that it was the responsibility of the trial team to communicate in an open and honest way with their TSC:

*‘You’ve got to take your dirty laundry to the TSC and get it cleaned. That’s what they’re there for. A lot of CIs don’t understand that the TSC is there to protect them and to help them resolve difficult issues. It’s fine when it’s all going hunky-dory, but a lot of CIs don’t understand, I think, that the trial oversight is their protection’*. [#39, CTU Director, RCT 7]

*‘What you need though is a very open culture to do that, because it’s the responsibility of the trial team to bring to the TSC those issues as they arise so you’ve got to, you’ve got to have a system and a culture which does that rather than hides things away.’* [#48, Sponsor representative]

These findings suggest some tension in the relationship between the trial team and the TSC. The TSC is perceived to be close to the funder and influential in determining whether a funder will withdraw funding, but the TSC is also perceived and described in the MRC guidelines as a committee of experts who guide trial conduct and help to ensure a successful trial.

## Discussion

This study is novel in its detailed exploration of relationships between stakeholders involved in trial oversight, and how these relationships impact the trial oversight process. We found evidence of good collaboration between CIs, TMs and CTUs, with clinicians and trialists learning from, and supporting, each other. Fundamental to this was openness and mutual respect. Between trial oversight committees, both collaboration and conflict were evident; conflict arose when there were differences of opinion regarding the best course of action and lack of clarity regarding lines of communication and responsibility. Differing stakeholder priorities reflected their perceptions of what was required from them professionally and could cause tension and conflict; for example, if a CI felt that others were undermining efforts to keep a trial open. Recognising, and respecting the value of, differing priorities among those involved in running trials is likely to be key to successful relationships between oversight stakeholders.

While CIs were perceived as highly invested in the trial succeeding (i.e. providing a reliable answer in a reasonable timeframe), even when the trial was in crisis, CTU staff were perceived as more objective and hence able to see if a trial faced intractable problems. As previous work has found, this independence was highly valued by funders as a way of enhancing the rigour of the trial [6]. In practice, this perceived objectivity depends on the relationship between the trial team member and the trial: a CTU-employed TM might also become highly invested in a trial, and indeed their professional standing might depend on it being well-managed. It is also unclear whether a CI’s personal investment in a trial is always problematic; ultimately, whether being highly invested in a trial is associated with poor trial methodology or conduct is an empirical question. In the context of trial oversight by multiple parties, the passion of one party (e.g. the CI) might be beneficial, with independent TSC members and the DMC playing a moderating role to ensure that good scientific conduct is maintained and participants’ wellbeing prioritised. However, a ceased trial in effect may be considered avoidable waste [19], and all efforts should be made to report and learn from the reasons that the trial ‘failed’, i.e. produced an unreliable result, or exceeded timeframe and/or budget. To ensure integrity in research [20], it is vital that lessons from such trials are incorporated into the future planning of trials and reflected in developments in trial oversight.

The role of the TM in acting as a primary point of contact and route for communication was important in ensuring the smooth running of a trial and coordinating the activities of trial oversight groups. This contrasts with the MRC guidelines [1], which currently list these roles as the PI’s; the MRC terminology should be updated to reflect current practise and the European Union Clinical Trials Regulation [21]. We found that clear communication channels between oversight groups were also needed if communication between the groups was not to break down; if this did occur, the CTU acting as arbiter could be helpful.

In this study, there was a lack of consensus regarding who held power within trial oversight. One funder had recently started selecting independent TSC members for the trials they funded, and this was perceived as problematic by some as it shifted the TSC’s loyalty from the trial to the funder. This chimes with the recent CTU survey [1]; respondents reported decisions made by the TSC have the potential to be overridden and expressed disquiet about this. In this study, the funder was perceived by some to be at the top of the power hierarchy within trial oversight, with the CI at the bottom, due to its role in financing the trial and potentially withdrawing funding. However, who has ultimate responsibility for shutting down a trial was contested, with sponsor, funder and TSC representatives all asserting that they could stop a trial. While this suggests lack of clarity about roles within trial oversight, it also points to variation in practice and power distribution depending on the funder. Our findings thus support the need for research into the effects of different ways of designing, conducting and overseeing evaluations of health and social care [22].

Perceived power and accountability affected relationships between stakeholders in trial oversight, with CIs in particular vulnerable to the threat of being called by a funder to a ‘monitoring meeting’ when the trial was not progressing well. A reaction to this threat was the CI putting a positive angle on trial progress when presenting to the TSC, which advises the funder. While this was seen as a natural consequence of the CI’s position vis-à-vis the funder, there were negative consequences identified for the CI and the rigour of the trial in that the expertise of the TSC would not be fully utilised. This highlighted a tension between the two roles of the TSC: influencing the funder and providing expert support to the CI and TMG. Research in organisational psychology has shown that communicating to a body perceived as having a higher status often involves trying to project a favourable image, and that this results in closed or incomplete communication [16]. In the context of trial oversight, guarded or incomplete exchanges of information could potentially damage the trust between oversight groups and reduce the effectiveness of trial oversight. This is the very behaviour that trial oversight aims to avoid: open and transparent communication from the outset could identify difficulties promptly and allow the team to find solutions to these challenges. A ceased trial can also be an opportunity for learning, and should be communicated as such. A shift in the communication pattern is, therefore, needed. This is likely to involve re-thinking the current power hierarchy in trial oversight, ensuring sufficient checks and balances to decrease the centralisation of power, and changing the behaviours and attitudes of both parties.

Findings from this study have clear implications for a revision of the MRC guidelines for GCP. We offer the evidence-based recommendations in Table 5 for future consultation. The role of the CTU and TM in delivering trials, and how they complement and support that of the CI, should be included in the new guidance. We also recommend that the terminology in the guidelines is reviewed and updated to reflect current usage, especially definitions of ‘CI’, ‘PI’ and ‘sponsor’. Agreeing on a common language would minimise the confusion identified by Conroy et al. [1].

**Table 5:** Recommendations regarding trial oversight

1. Led by their Chairs, trial oversight committees must foster a culture of openness and mutual respect, recognising and drawing upon the skills of all trial oversight committee members. To ensure optimal decision-making and problem-solving, committee conduct should be respectful of all voices and Chairs should actively seek opinions from all members	
2. Recognise the partnership role of CTUs and TMs in managing a trial and supporting the CI in decision-making, management and achieving deliverables	
3. The differing priorities of trial stakeholders in overseeing and delivering the trial should be explicitly stated, considered and, where necessary, realigned to the shared priority across stakeholder groups to produce a good-quality trial that informs practice. Guidance and best practice on resolving differences should be shared and could be collated and hosted by CTUs and funders	
4. Clear lines of communication between oversight groups should be established in advance of the trial starting, documented in the trial Charter, shared between stakeholders, and maintained. From the trial outset, the frequency of oversight meetings should be considered and agreed. The frequency should be regular enough to be responsive to challenges and implement trial oversight decisions promptly, while allowing for extraordinary meetings in the event of challenges	
5. Ensure a primary, single point of contact for the trial and coordinate communication with trial oversight stakeholders	
6. Consider and agree before trial initiation who will act as arbiter if/when needed; the CTU may or may not play this role	
7. Consult with stakeholders to determine the full implications of funders appointing independent TSC members to trials, and agree an approach to this issue	
8. Clarify the roles and responsibilities of all those involved in trial oversight, and make stakeholders aware of these. This includes the different reasons for shutting down a trial, and which oversight bodies might do so in which circumstances. Each trial should consider these from the outset. Discuss before trial initiation the information needs of different stakeholders and communicate efficiently and in a timely manner as needed	
9. Be aware of how the threat of monitoring meetings, or of closing trials, can negatively impact trial conduct and relationships, especially the way a CI or TMG might present the trial to the TSC. It might be of benefit to identify risks to the trial from the outset, and report on these at each meeting	
10. Acknowledge how the close relationship between TSC and funder and the threat of the latter withdrawing funding is in tension with the role of the TSC in providing expert support to the TMG. Power hierarchies between committees can restrict the effectiveness of trial oversight, so efforts should be made to decentralise power	

*CI* Chief Investigator, *CTU* Clinical Trials Unit, *TMG* Trial Management Group, *TSC* Trial Steering Committee

This study has strengths and limitations which should be considered in interpreting the transferability of our findings. We triangulated multiple ethnographic methods, which allowed an in-depth exploration of trial oversight practices in action from several perspectives. While the DAMOCLES study [4] relied on recall of group decision-making through interviews, our observation of oversight meetings illustrated the ways in which relationships actually impact on the conduct and quality of trial oversight. We used overt, rather than covert, participant observation, which is subject to possible observer effects such as social desirability bias. Unobserved trial oversight might, therefore, be subject to more tensions and difficulties than we report. The trials in this study were academic-led and publicly funded trials in the UK; it would be interesting to explore trial oversight further within industry-coordinated trial settings. The tripartite trial oversight structure involving independent TSC members commonly employed in the UK is not widely used in other countries. We selected only eight trials, all phase III RCTs, which cannot be representative of all RCTs, although parallels exist between trials. Furthermore, we selected trials experiencing challenges to give added insight into the behavioural patterns of trial oversight groups. However, an interesting area for future research would be to compare our findings with the trial oversight of trials which are not undergoing such difficulties, as experiences and opinions may differ.

Further research is needed to expand the evidence base regarding trial oversight. In particular, the behaviour of individuals within trial oversight groups (intragroup behaviours) has not yet been explored. In DAMOCLES, intragroup factors, such as biased or overly directive leadership and an expression of a limited range of opinions during group discussion ‘groupthink’, were found to impact the productivity of DMC trial oversight meetings [4], but the presence of these behaviours in real-life TSCs and TMGs has not yet been explored. The role of PPI in trial oversight also needs further examination; our findings regarding PPI will be reported separately.

## Conclusions

Relationships between the stakeholders involved in trial oversight are central to the process and quality of that oversight. Findings from this study have important implications for revising GCP guidelines and trial management for RCTs internationally.

## Abbreviations

CTU: Clinical Trials Unit
DMC: Data Monitoring Committee
GCP: Good Clinical Practice
MRC: Medical Research Council
NHS: National Health Service
NIHR: National Institute for Health Research
RCT: Randomised controlled trial
TM: Trial manager
TMG: Trial Management Group
TSC: Trial Steering Committee
